# Statistical Learning for Speech Segmentation: Age-Related Changes and Underlying Mechanisms

**DOI:** 10.1037/pag0000292

**Published:** 2018-09-24

**Authors:** Shekeila D. Palmer, James Hutson, Sven L. Mattys

**Affiliations:** 1 Department of Psychology, University of York

**Keywords:** statistical learning, speech segmentation, aging, working memory

## Abstract

Statistical learning (SL) is a powerful learning mechanism that supports word segmentation and language acquisition in infants and young adults. However, little is known about how this ability changes over the life span and interacts with age-related cognitive decline. The aims of this study were to: (a) examine the effect of aging on speech segmentation by SL, and (b) explore core mechanisms underlying SL. Across four testing sessions, young, middle-aged, and older adults were exposed to continuous speech streams at two different speech rates, both with and without cognitive load. Learning was assessed using a two-alterative forced-choice task in which words from the stream were pitted against either part-words, which occurred across word boundaries in the stream, or nonwords, which never appeared in the stream. Participants also completed a battery of cognitive tests assessing working memory and executive functions. The results showed that speech segmentation by SL was remarkably resilient to aging, although age effects were visible in the more challenging conditions, namely, when words had to be discriminated from part-words, which required the formation of detailed phonological representations, and when SL was performed under cognitive load. Moreover, an analysis of the cognitive test data indicated that performance against part-words was predicted mostly by memory updating, whereas performance against nonwords was predicted mostly by working memory storage capacity. Taken together, the data show that SL relies on a combination of implicit and explicit skills, and that age effects on SL are likely to be linked to an age-related selective decline in memory updating.

For new language learners, segmenting fluent speech into its component words poses a significant computational challenge. In natural speech, words are rarely separated by pauses and, in the absence of lexical support, the speech signal offers few reliable cues to enable identification of word boundaries. However, infants appear to be equipped with a powerful learning mechanism that helps them overcome this challenge with remarkable ease. Infants as young as 8 months are sensitive to the statistical properties of speech, such that they are able to extract words based solely on transitional probabilities between syllables within a continuous speech stream (e.g., Saffran, Aslin, & Newport, 1996). However, language learning is not confined to infancy and, while numerous studies have shown that statistical learning (SL) remains robust in young adults (e.g., Saffran et al., 1996, Saffran, Newport, Aslin, Tunick, & Barrueco, 1997), very little is known about how SL is affected by cognitive aging.

A reason why it is difficult to predict how SL abilities might change over the life span is that there is little agreement with regard to the mechanisms that underpin this form of learning. Since SL operates in infants, and even nonhuman species (e.g., Hauser, Newport, & Aslin, 2001; Toro & Trobalón, 2005), it is usually assumed to be an automatic learning mechanism that requires few cognitive resources (e.g., Fiser & Aslin, 2001; Saffran et al., 1996, 1997; Turk-Browne, Jungé, & Scholl, 2005, but see Toro, Sinnett, & Soto-Faraco, 2005). This assumption is generally supported by studies showing that SL can occur implicitly, without either intention to learn (Fernandes, Kolinsky, & Ventura, 2010; Turk-Browne et al., 2005) or explicit knowledge of the information acquired (Kim, Seitz, Feenstra, & Shams, 2009; Turk-Browne et al., 2005). From this perspective, one might expect that SL abilities should be relatively resilient to cognitive aging. Indeed, it is often argued that, unlike working memory, executive control, and processing speed, which show age-related decline, implicit and automatic processes remain comparatively stable throughout life (e.g., Fleischman, 2007; Fleischman, Wilson, Gabrieli, Bienias, & Bennett, 2004; Jennings & Jacoby, 1993; Mitchell, 1989).

Consistent with this view, evidence suggests that older adults maintain sensitivity to statistical regularities in the visual domain. For example, Campbell, Zimerman, Healey, Lee, and Hasher (2012) presented groups of younger (17–25 years) and older (59–81 years) adults with two interleaved streams of pictures and asked them to perform a 1-back task on one of the streams while ignoring the other. Unbeknownst to the participants, the pictures in both streams were arranged into triplets, and the pictures within each triplet always occurred in the same sequential order. After exposure, triplet learning was tested using a speeded detection task, in which faster response time across triplet position was taken as evidence of associative knowledge of the triplets. The results indicated not only that older adults learned the triplets in the attended stream, but also that, unlike their younger counterparts, they showed evidence of learning triplets in the unattended stream. This led Campbell et al. (2012) to conclude that older adults could in fact be *more* sensitive to statistical regularities than younger adults.

However, there are reasons to believe that older adults might show a deficit in SL in some circumstances. While implicit processes remain more stable throughout the life span than explicit processes, not all forms of implicit learning are immune to aging. Most relevant to SL are findings relating to the serial reaction time (SRT) task, which involves learning statistical relationships between events. In the standard version of the SRT task (Nissen & Bullemer, 1987), participants are presented with an array of boxes on a computer screen and, on each trial, a stimulus appears in one of the boxes. The task is to press a key corresponding to that box as quickly as possible. In experimental blocks, the stimuli follow a predictable repeating sequence across trials. Implicit learning of the sequence is assessed by comparing response times in the experimental blocks to those in control blocks, where the sequence is random. In a review of studies investigating age-related changes in SRT performance, Howard and Howard (2013) noted that those examining simple deterministic regularities do not usually reveal age-related deficits, whereas those using probabilistic versions of the SRT task do (e.g., the alternating serial reaction time task [ASRT]). Moreover, the magnitude of the age deficit appears to depend on the size of the lag between predictable events in the sequence. For example, for lag-2 sequences (e.g., 1r2r3r4r1r2r3r4, where 1–4 represent a predictable sequence of events, and r is any of these events randomly inserted between predictable events), older adults show some evidence of learning, but they learn less well and more slowly than younger adults (Howard & Howard, 1997). For lag-3 sequences, which contain two random events inserted between two predictable events (e.g., 1rr2rr3rr4rr1rr2rr3rr4), young adults continue to show evidence of learning, but older adults do not (Howard et al., 2004).

Another reason to suspect that older adults may be disadvantaged in SL tasks relative to younger adults is that it has been shown that SL depends partly on active working memory processes. For example, in a sample of young adults, Palmer and Mattys (2016) observed that SL performance was inversely related to speech rate: the slower the stream, the better SL performance. However, the benefit associated with slowing down the rate was eliminated when participants were asked to perform a visual 2-back task while listening to the stream. This result suggests that SL performance is supplemented by an active maintenance mechanism that operates more effectively when processing time is increased, but is disrupted when central resources are depleted by cognitive load. Since the level of disruption was independent of load type (phonological vs. nonphonological 2-back tasks), the authors concluded that this mechanism involved domain-general executive resources and, in particular, working memory updating. They argued that an active updating mechanism may contribute to SL by removing and replacing erroneous syllable groupings held in working memory. Since working memory updating is known to show marked age-related decline (De Beni & Palladino, 2004; Fiore, Borella, Mammarella, & De Beni, 2012; Van der Linden, Brédart, & Beerten, 1994), this process may be less efficient in older adults, thereby leading to an age-related decrease in SL performance.

Finally, there is some evidence that another form of SL, namely artificial grammar learning, shows some age-related decline (e.g., Lukács & Kemény, 2015; Midford & Kirsner, 2005; Schwab at al. 2016). In a recent life span study, Lukács and Kemény (2015) found that grammar learning performance, along with performance on an SRT task, tended to peak in early adulthood, and then declined gradually until the age of around 65, when decline accelerated significantly. Although the overall developmental pattern of decline was similar for the grammar learning and SRT tasks, performance on these two tasks was uncorrelated. The magnitude of the age effect and the timing at which age-related changes in performance occurred also differed between tasks. Lukács and Kemény (2015) note that although both tasks measure implicit learning, the contribution of working memory and explicit process is unclear, and that performance on the tasks is likely to rely on different combinations of memory systems. Therefore, in order to predict how performance on SL might be affected by age-related cognitive decline, it is necessary to gain a better understanding of the mechanisms that underlie SL performance.

Consequently, the aim of the current study was twofold. First, we aimed to assess the effect of age on speech segmentation by SL and, second, we wanted to gain further insight into the processes that underlie performance in this task. Following Palmer and Mattys (2016), we assume that SL in adults includes an implicit (or incidental) component, which operates automatically, but that performance can be boosted by an explicit (or active) component, which relies on working memory. In that context, and that of the literature reviewed above, two scenarios are considered.

On the one hand, given that older adults generally show a decline in working memory resources, their SL performance might be poorer than that of younger adults. This age difference should be exacerbated when working memory resources are taxed by a cognitive load task. Furthermore, slowing down the speech rate should benefit older adults less than younger adults since older adults may be less able to capitalize on the increased processing time afforded by the slower rate. In fact, slow speech could even have a detrimental effect on SL in older adults, as reduced working memory may make it more difficult to counteract decay of the to-be-bound syllables.

On the other hand, it is possible that the magnitude of the cognitive load and/or rate effect will not differ substantially between younger and older adults. This outcome would be expected if it turns out that SL relies more heavily on incidental learning mechanisms than Palmer and Mattys’ (2016) data suggest and if the kind of implicit learning involved in SL is resilient to age-related cognitive decline.

In the following study, young, middle-aged, and older adults completed four SL speech segmentation tasks across four separate testing sessions. The middle-aged group was included to give an indication of the trajectory of SL abilities over the life span. This is particularly relevant since age deficits in ASRT tasks are already observable in adults between 34 and 53 years of age (Feeney, Howard, & Howard, 2002). By contrast, life span data reported by Lukács and Kemény (2015) suggest that performance on other SL tasks, such as SRT and grammar learning, does not show clear age-related decline until participants reach their mid-60s. Across the four testing sessions, we manipulated speech rate and cognitive load, with each participant performing a speech segmentation task on streams played at two different rates (normal vs. slow), both with and without cognitive load (a concurrent visual 2-back task).

Learning was assessed using a two-alternative forced-choice task that included two types of trials: (a) word-partword (WD-PW) pairs, in which a word from the stream was pitted against a sequence that occurred across word boundaries, and (b) word-nonword (WD-NW) pairs, in which a word from the stream was pitted against a sequence that never occurred in the stream. Both trial types are commonly used as measures of SL, but they are rarely included within the same experiment, and the extent to which they measure the same underlying computations is unclear. Specifically, for WD-PW trials, the foils (part-words) are heard during the learning phase, which is not the case for WD-NW trials. Therefore, at the very least, the WD-PW trials should constitute a case of more difficult discrimination, and thus a more stringent test of SL. Given previous research showing that age-related deficits in SRT performance emerge only when the task is sufficiently complex (e.g., Howard & Howard, 2013), we predict that any age effects in SL will be most visible when the discrimination test is complex (i.e., on WD-PW trials). Furthermore, since performance on these trials is likely to depend on participants having acquired more precise representations of the words in the stream, it follows that performance on WD-PW trials should be more strongly related to higher level executive function.

Finally, in order to gain greater insight into the processes that underlie performance on WD-PW and WD-NW trials and to help account for any age differences observed, participants completed tests of working memory capacity and processing speed. In particular, we wanted to test Palmer and Mattys’ (2016) proposal that working memory updating might support SL. To determine whether SL is associated specifically with working memory updating, rather than speed of processing and/or working memory capacity, in general, performance on different measures of working memory and processing speed were entered into a regression analysis as predictors of SL, separately for the WD-PW trials and the WD-NW trials.

## Method

### Participants

Ninety-six native British English speakers participated in the experiment. They included 32 young adults aged between 18 and 25 years ($\bar{x}$ = 21), 32 middle-aged adults aged between 40 and 50 years ($\bar{x}$ = 45), and 32 older adults aged between 60 and 81 years ($\bar{x}$ = 68). Most of the young adults were students at the University of York, while participants in the other age groups consisted of a combination of university staff and people recruited from the local community. Average audiometric pure-tone thresholds of each age group are shown in the online supplemental materials. Approval for performing the testing described below was granted by the University of York Departmental Ethics Committee (Application 215) on March 13, 2015.

### Neuropsychological Testing

This study was conducted as part of a larger aging project in which all participants completed a battery of neuropsychological tests. Within the context of the present experiment, the variables of interest were those relating to working memory and executive function, including forward and backward digit spans, working memory updating, and inhibition (measured using a Stroop task). Additionally, processing speed was measured using the Digit Symbol-Coding and Symbol Search subtests of the Wechsler Adult Intelligence Scale (Tulsky, Zhu, & Ledbetter, 1997). Full descriptions of the working memory tasks are provided in the online supplemental materials. The Mini Mental State Exam (MMSE; Folstein, Folstein, & McHugh, 1975) was administered to the older age group to screen for signs of abnormal cognitive decline. All participants scored above the standard cutoff of 24 points on the MMSE.

### Materials

#### SL familiarization streams

For the familiarization phase, four artificial languages were created, each made of a different pool of syllables (languages are provided in the online supplemental materials). The syllables of each language were used to generate two counterbalanced streams (Stream A and Stream B). Each stream was made up of two trisyllablic (e.g., rebufi) and two quadrisyllabic (e.g., lasokachu) “words” concatenated in a pseudorandom order such that there were no immediate repetitions. We used words of different lengths because we wanted to ensure that participants did not become familiar with the rhythmic properties of the stream after the first session. Within a language, the Stream B words were created by reorganizing the syllables of the Stream A words such that the Stream B words consisted of syllables occurring across word boundaries in Stream A and vice versa. Specifically, the trisyllabic words in Stream B were created by concatenating the last two syllables of one of the quadrisyllabic words in Stream A and the first syllable of one of the trisyllabic words in Stream A. The quadrisyllabic words in Stream B were created by concatenating the last two syllables of one of the trisyllabic words in Stream A and the first two syllables of one of the quadrisyllabic words in Stream A.

Within a stream, each word was played 108 times resulting in a total of 432 words per stream. The transitional probability between the syllables forming a word was 1.00 and the transitional probability between syllables spanning word boundaries was 0.33. The streams were synthesized using the text-to-speech MBROLA software (Dutoit, Pagel, Pierret, Bataille, & van der Vrecken, 1996) with an English male diphone database. Synthesis ensured that there were no acoustic boundary cues within the speech streams and that the main cues for inferring word boundaries were the relative between-syllable transitional probabilities within and across words. Each stream was generated at two different rates. The normal rate was 4.17 syllables per second and the slow rate was 2.27 syllables per second. The normal rate was comparable to the average rate in most SL studies. The slow rate approximated the rate of slightly slower-than-average clear speech. The duration of each stream was 6 min for the normal rate and 10 min 50 s for the slow rate.

In order to verify that the normal and slow rates were equally intelligible and that intelligibility was not unduly influenced by age, an intelligibility test was conducted. Twelve young adults (18–25 years) and eight older adults (60–85 years), who did not take part in the main study heard the 32 trisyllabic and quadrisyllabic strings used as words in the eight speech streams. Strings were played one at a time. Each participant heard one block of 16 strings played at the normal rate, and the other 16 strings played at the slow rate in a second block. The strings in each block and the order in which the blocks were presented were counterbalanced between participants. On each trial, the syllable string was played twice. A question mark then appeared on the screen, prompting participants to repeat the syllable string out loud. The participant controlled the pace of the test by pressing the space bar to hear each string. Responses were audio recorded and scored offline by the experimenter by calculating the proportion of consonants and vowels produced correctly.

An analysis of variance (ANOVA) with age group (young, older), segment (consonants vs. vowels), and stream rate (normal, slow) showed an effect of segment, with vowels (95%) perceived more accurately than consonants (92%), *F*(1, 18) = 23.76, *MSE* = .017, *p* < .001, but there was no significant difference in intelligibility between the young (93%) and older (95%) groups, *F*(1, 18) = 1.84, *MSE* = .003, *p* = .19, or between the normal (94%) and slow (94%) stream rates, *F*(1, 18) < 1. None of the interaction terms reached significance (all *p*s >.20). Thus, intelligibility was high across all conditions and not significantly influenced by rate or age.

#### SL recognition test

The recognition test for the SL task was a two-alternative forced-choice task composed of 24 pairs. The 24 pairs included eight word versus part-word trials (WD-PW), eight word versus nonword trials (WD-NW), and eight part-word versus nonword trials (PW-NW). For WD-PW trials, a word from the familiarization phase was paired with a part-word, that is, a word from the other stream of the same language. Part-words were named as such because they occurred across word boundaries in the familiarization stream. For WD-NW trials, a word from the familiarization phase was paired with a nonword. Nonwords were syllable strings created using syllables heard in the familiarization stream, but concatenated in a scrambled order so that any two consecutive syllables never occurred in that order in the familiarization stream. For PW-NW trials, a part-word was paired with a nonword. The PW-NW trials were included as filler trials to ensure that part-words, nonwords, and words appeared equally often during the test phase. Within each trial type, each syllable string appeared twice, each time paired with a different string. Across all trials, syllable strings were only paired with strings of the same length (i.e., a quadrisyllable was never paired with a trisyllable). The order in which the word pairs were presented was randomized between participants.

#### Cognitive load task

For the cognitive load conditions, a 2-back task was created. This consisted of rapid serial presentation of visual stimuli displayed concurrently with the speech stream. The stimuli were 86 line drawings taken from Kroll and Potter (1984), which were all novel, meaningless, and nonnameable shapes. Half of the shapes were rotated 30° to the left and the other half were rotated 30° to the right. The 2-back repetition trials always involved a change in orientation: if the first shape was rotated to the right, the repetition was rotated to the left, and vice versa. Each shape was displayed for 750 ms. Shapes were separated by 250 ms of blank screen. The pace was the same regardless of the speech rate. It was chosen to avoid synchrony between the onset of the visual stimuli and the onset of words or syllables in the stream. The total number of shapes displayed depended on the duration of the speech stream. The normal rate included 481 shapes with 78 2-back pairs, whereas the slow rate included 864 shapes with 140 2-back pairs.

### Design and Procedure

The experiment followed a 2 × 2 design with cognitive load and speech rate as within-subjects variables. Each participant completed the following four conditions: (a) normal rate with no cognitive load, (b) normal rate with cognitive load, (c) slow rate with no cognitive load, and (d) slow rate with cognitive load. In each condition, participants were tested using a different language. Each of the four conditions was completed during one of four individual testing sessions. The session timings were arranged in accordance with the participant’s availability, with the constraint that there should be at least 48 hr and no more than 2 weeks between two consecutive sessions. The order in which the conditions were completed was counterbalanced between participants. Each session lasted approximately one hour and consisted of an SL task and a subset of neuropsychological tests (detailed above). Participants always completed the SL task before performing the neuropsychological tests. All participants completed the audiometric test at the start of Session 1. Hearing thresholds were tested for 250, 500, 1000, 2000, 4000, and 8000 Hz.

The experiment took place in a sound-attenuated booth. Stimuli were played over headphones. Several steps were taken to ensure that the sound level was appropriate for each individual participant. First, each participant performed an audiometric test before taking part in the experiment. Participants were then played nonsense trisyllabic sequences over the headphones and asked whether the sound level was comfortable and whether they could hear the syllable sequences clearly. Sound level was adjusted accordingly. Finally, participants performed a syllable intelligibility test in which they heard 16 syllables (four from each of the four languages). After each syllable, participants were asked to repeat what they heard. If a syllable was repeated incorrectly, the experimenter corrected it and replayed the syllable. One participant in the older age group was eliminated and later replaced due to poor performance in the syllable test.

#### Familiarization phase

Participants were told that they would hear an artificial language and that they should try to discover what the words of the language were. To ensure that participants understood the instructions and knew what to expect, they were always played a sample stream for 30 s prior to starting the familiarization phase. The sample stream was played at the same rate as the real subsequent familiarization stream. It included a combination of syllables from each of the four languages, but did not include words from any of them. In the cognitive load conditions, participants also practiced the cognitive load task while listening to the sample stream. During the first session, participants were shown a sample trial of the test phase after hearing the sample stream so that they understood the format of the test. Before hearing the experimental stream, participants were informed that the stream would be made up of artificial words that were three and four syllables in length. However, they were not told how many words were in the stream or the proportion of trisyllables and quadrisyllables. Since our design was within subjects, these procedures were employed to minimize the impact of “novelty” during the first session, and ensure that the participant’s expectations at the start of the first session were as similar as possible to those at the start of the later sessions.

**Figure 1 fig1:**
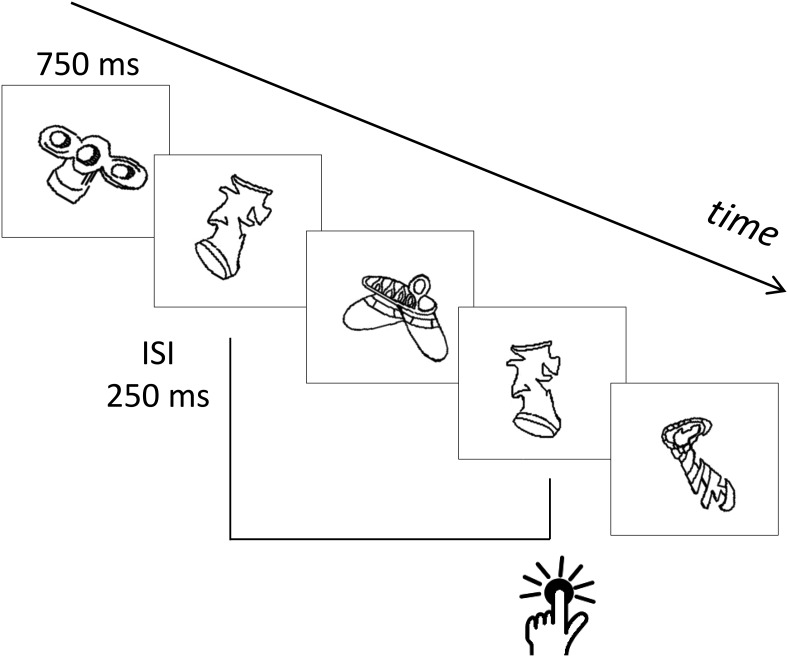
Illustration of the cognitive load (2-back) task.

In the cognitive load (2-back) conditions, participants were instructed to press the space bar every time they saw a shape that was the same as the shape that appeared two trials before (Figure 1). Participants were informed that the shape might have a different orientation on its second presentation.[Fig-anchor fig1]

#### Recognition test

For each trial in the recognition task, the two strings of a pair were presented both visually and auditorily. The two strings were separated by a 500-ms silent interval and were played at the same rate as the speech stream heard during familiarization. As the first string was played, its orthographic transcription appeared on the left-hand side of the computer screen for 1,500 ms and disappeared before the second string was played. The orthographic transcription of the second string then appeared on the right-hand side of the screen as the second string played, and stayed for 1,500 ms. Then, both transcriptions appeared again, simultaneously, and remained on the screen until the participant responded. Supplementing the auditory strings with visual support was intended to prevent memory decay between the first and second strings, which could have excessively affected the older participants. Participants were asked to indicate which syllable string had occurred more frequently in the artificial language using the left or right shift key for the first or second syllable string in each pair, respectively. The next pair was presented 1,000 ms after the participant’s response.

## Results

### SL Task

**Figure 2 fig2:**
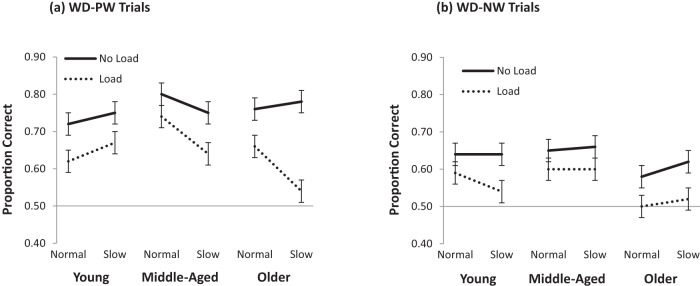
Mean proportion of correct responses in the normal and slow speech rate conditions, under no load and cognitive load for young, middle-aged, and older adults collapsed across trial types for word-partword (WD-PW) trials (a), and for word-nonword (WD-NW) trials (b). Error bars represent the standard error of the mean.

Mean accuracy in the recognition test is presented in Figure 2. The data were analyzed using mixed effects logistic regression models with age group (young, middle-aged, older), cognitive load (no load, load), stream rate (normal, slow), and trial type (WD-PW, WD-NW trials) as fixed factors. The random structure included random intercepts by participant and item, and random slopes for load by participant, rate by participant, load by item, and rate by item. The load and rate variables were centered with no load and slow rate coded as −1 and cognitive load and normal rate coded as 1. The fixed factors and the interaction terms were added incrementally, starting from a base model that included only the random terms. Improved fit was assessed using the likelihood ratio test.[Fig-anchor fig2]

Cognitive load significantly affected performance, β = −.24, *SE* = .04, χ^2^(1) = 26.49, *p* < .001, with participants performing worse in the load (.60) than no load (.70) conditions. Trial type also predicted performance, β = .54, *SE* = .06, χ^2^(1) = 89.72, *p* < .001, with better performance on the WD-NW (.70) than WD-PW trials (.60). Neither stream rate, β = .03, *SE* = .04, χ^2^(1) = .54, *p* = .46, nor age group, β = .07, *SE* = .06, χ^2^(1) = 1.53, *p* = .21, improved fit over the base model. Several two-way and three-way interactions were found, but a significant four-way interaction, β = −.12, *SE* = .07, χ^2^(1) = 27.65, *p* = .004, prompted us to split the analyses by trial type (WD-PW vs. WD-NW), which was the strongest predictor of performance (as indicated by β) and represented a more (WD-PW) versus less (WD-NW) stringent test of SL.

Consistent with the main analysis, performance on the WD-PW trials was worse under load than no load, β = −.18, *SE* = .05, χ^2^(1) = 11.44, *p* < .001, and it was unaffected by rate, β = −.004, *SE* = .05, χ^2^(1) = 0.01, *p* = .90. This time, however, there was some indication that age group affected performance, β = .11, *SE* = .12, χ^2^(1) = 0.06, *p* = .07: older adults were marginally worse than younger adults, β = .12, *SE* = .07, χ^2^(1) = 3.00, *p* = .08, and significantly worse than middle-aged adults, β = .33, *SE* = .10, χ^2^(1) = 9.39, *p* = .002. The young and middle-aged adults did not differ from each other, β = −.11, *SE* = .14, χ^2^(1) = .61, *p* = .43. Poorer performance of the older age group was due, in part, to older adults performing at chance level under cognitive load (*p* = .71). This contrasted with the younger adults, who were marginally above chance (*p* = .06), and the middle-aged adults who were significantly above chance (*p* = .002). Performance in the no-load condition was significantly above chance for all three groups (all *p*s < .001). No significant interactions were observed.

For the WD-NW trials, cognitive load was a significant predictor of performance, β = −.31, *SE* = .04, χ^2^(1) = 31.99, *p* = .001, but stream rate, β = .08, *SE* = .05, χ^2^(1) = 2.61, *p* = .11, and age group, β = .02, *SE* = .07, χ^2^(1) = .06, *p* = .81, were not. A significant three-way interaction, β = −.10, *SE* = .05, χ^2^(4) = 14.56, *p* = .006, showed age-dependent patterns of interaction between load and rate. While load and rate did not interact in either the young or the middle-aged groups (β = −.03, *SE* = .07, χ^2^(1) = 0.15, *p* = .70, and β = .05, *SE* = .08, χ^2^(1) = 0.41, *p* = .52, respectively), they did in the older group, β = 0.17, *SE* = .07, χ^2^(1) = 5.35, *p* = .02. This pattern was driven by poorer performance by the older adults in the slow rate condition under cognitive load (SR-CL), and indeed, this was the only condition that did not significantly differ from chance (*p* = .25; all others, *p* < .01). Significant interactions between age group and cognitive load, β = .11, *SE* = .05, χ^2^(1) = 4.28, *p* = .04, and between age group and rate, β = −.12, *SE* = .06, χ^2^(1) = 4.53, *p* = .03, confirmed this interpretation, with an effect of age group in the SR-CL condition, β = .30, *SE* = .10, χ^2^(1) = 8.63, *p* = .003, but not in the other three conditions (all *p*s >.10). Analyses of simple effects revealed that the older adults were worse in the SR-CL condition than both the young adults, β = .30, *SE* = .09, χ^2^(1) = 9.54, *p* = .002, and the middle-aged adults, β = .44, *SE* = .20, χ^2^(1) = 4.83, *p* = .03.

In summary, we found that cognitive load impaired speech segmentation performance, but, overall, speech rate did not. Although no main effect of age group was observed, the older group performed worse than the young and middle-aged groups and it was the only group that did not perform above chance under cognitive load on the WD-PW trials. On the comparatively easier WD-NW trials, the older group performed worse than the other groups in the SR-CL condition only, where, again, performance was not significantly above chance. The fact that there were no significant differences between young and middle-aged adults in any of the conditions indicates that speech segmentation by SL remains robust in middle age.

### Performance in the 2-Back Task

Hit rates, false alarm rates, and *d*′ scores for the 2-back task are shown in Table 1.^1^ The hit rate was calculated as the number of correct responses to 2-back repetitions divided by the total number of 2-back repetitions in the stream. The false alarm rate was calculated as the total number of incorrect responses to nonrepeated stimuli divided by the total number of nonrepeated stimuli. Since *d*′ scores are based on hit and false alarm rates aggregated over a large set of individual responses, the *d*′ scores were analyzed through a two-way mixed ANOVA with stream rate (normal, slow) as the within-subjects variable and age group (young, middle-aged, older) as the between-subjects variable. This analysis showed that stream rate did not affect discrimination performance, *F*(1, 91) = 2.73, *MSE* = .38, *p* = .10, but there was a marginal difference in discrimination performance according to age group, *F*(2, 91) = 2.58, *MSE* = .70, *p* = .08.[Table-anchor tbl1]

### Neuropsychological Measures

Mean performance in the neuropsychological tests for each age group is shown in Table 2 (see Footnote 1). Average hearing thresholds are also included. Table 3 shows the correlations between performance on each of the neuropsychological tests and performance in the SL task, both overall, and split by load and trial type.[Table-anchor tbl2][Table-anchor tbl3]

In order to determine how well individual differences in working memory, executive function, and processing speed predicted SL performance when controlling for age and hearing, hierarchical multiple regression analyses were performed. Given our hypothesis that performance on WD-PW trials may be more dependent on higher level executive function than performance on WD-NW trials, we ran separate regression analyses for WD-PW and WD-NW trials. In both analyses, age and hearing were entered as predictors in the first block of the regression. Forward digit span, backward digit span, working memory updating, Stroop, and processing speed were entered in a second block. In order to minimize any outlier-related bias in the regression models, we eliminated extreme scores from the data by removing participants whose performance was more than 2 *SD* above or below the mean on any of the neuropsychological tests. This resulted in the loss of four participants: one from the middle-aged group and three from the older group.

In the WD-PW condition (Table 4), Block 1 was not significant, *R*^2^ = .05, *p* = .09, but Block 2 was, *R*^2^ = .19, *p* = .02, with working memory and processing speed increasing the explained variance from 5% to 19%, Δ*R*^2^ = .13, *p* = .03. In Block 2, working memory updating was the only significant predictor, β = .25, *p* = .03, showing that better working memory updating was associated with better performance. The other variables did not have a significant unique contribution.[Table-anchor tbl4]

The results of the WD-NW regression analysis are shown in Table 5. As in the previous analysis, hearing and age did not predict performance in Block 1, *R*^2^ = .01, *p* = .78. Again, the second block was significant, *R*^2^ = .16, *p* = .04, with working memory and processing speed increasing the explained variance from 1% to 16%, Δ*R*^2^ = .16, *p* = .01. This time, however, forward digit span was the only significant predictor of performance, β = .30, *p* = .04, showing that a larger working memory storage capacity was related to better performance on WD-NW trials. The other variables did not have a significant unique contribution.[Table-anchor tbl5]

In sum, age, hearing, and processing speed did not predict SL performance in either regression analysis. Working memory resources contributed to SL, but, critically, the type of resources involved seemed to depend on the nature of the computation performed. For the WD-NW trials, SL performance was related to general working memory storage capacity, with forward digit span accounting for 30% of the variance on these trials. In contrast, for the WD-PW trials, performance was related to working memory updating, which accounted for 26% of the variance. This shows that performance on WD-PW trials is more dependent on higher level executive function, and specifically, the ability to actively update the content of working memory. Interestingly, performance on WD-PW trials was not predicted by forward digit span, or by our other measures of executive function (backward digit span or inhibition [Stroop]).

## Discussion

The purpose of the current study was to (a) investigate the effect of aging on speech segmentation by SL, and (b) gain further insight into the mechanisms that underlie this ability. In relation to our first question, the results indicate that, in general, SL is remarkably resilient to age-related decline. The performance of the middle-aged adults did not differ from that of young adults in any of the conditions tested; in fact, it was numerically slightly better. Perhaps more surprisingly, across all trials, SL in the no-load condition was almost equivalent in the young and older adults. At first glance, this suggests that SL is akin to other forms of implicit learning, to the extent that it is characterized by relative stability across the life span, compared with explicit forms of learning (e.g., paired-associate learning), which tend to show more marked age-related decline (e.g., Naveh-Benjamin, 2000; Naveh-Benjamin, Guez, Kilb, & Reedy, 2004; Naveh-Benjamin, Hussain, Guez, & Bar-On, 2003). However, when the data were examined more closely, evidence of age-related decrement in SL was visible in the older group under some circumstances. Specifically, age effects emerged in the more challenging conditions. A detailed examination of performance across these conditions provides insight into the mechanisms that underlie SL.

Despite the generally comparable performance across age groups, the older adults tended to perform less well than the other groups on the more difficult WD-PW trials. In contrast, performance on the WD-NW trials was largely unaffected by age. Since the part-words used as foils on WD-PW trials were present as such in the stream, it can be argued that WD-PW trials constitute a stronger test of SL than WD-NW trials, in which the nonword foils were never heard before. Indeed, succeeding on WD-PW trials is contingent on acquiring distinct and precise representations of the words in the stream, whereas succeeding on WD-NW trials only requires a general gist of familiar-sounding sequences. Therefore, although older adults appeared unimpaired when performance was considered across all trial types, age deficits were visible when more sensitive measures of SL were used. Likewise, the older adults were the only group who did not perform above chance on the WD-PW trials in the cognitive load conditions. The young and middle-aged groups both continued to learn under cognitive load, albeit to a lesser extent than in the no-load conditions. These findings suggest that older adults have more difficulty using transitional probabilities to form accurate word representations than younger adults, and that this age-related deficiency is exacerbated under cognitive load.

The fact that older adults failed to learn under cognitive load is consistent with the hypothesis that working memory resources contribute to SL. Since working memory typically shows some age-related decline (e.g., Bopp & Verhaeghen, 2005; De Beni & Palladino, 2004; Fiore et al., 2012; Van der Linden et al., 1994), older adults are likely to have fewer resources available to cope with the dual-task demand. Palmer and Mattys (2016) previously suggested that working memory updating may be particularly important for SL. This hypothesis was supported by analysis of the neuropsychological test data which revealed that performance on WD-PW trials was predicted by working memory updating; participants with stronger memory updating scores tended to perform better on WD-PW trials. Since the older adults performed worse on the working memory updating task than the other age groups, this could explain, at least in part, why they had more difficulty with the WD-PW trials.

There are at least two ways in which working memory updating might benefit SL performance. One possibility is that updating benefits SL indirectly. Here, participants who are better at updating are necessarily better at coping with the cognitive load task since the 2-back is essentially a memory updating task. However, it seems unlikely that this is the only way in which updating benefits SL. As shown in Table 3, the correlation between working memory updating and SL performance was comparable in the load and no-load conditions for WD-PW trials. It was also the case that updating scores predicted SL performance only on WD-PW trials. They did not significantly predict SL performance on WD-NW trials, for which short-term memory storage capacity, as indexed by forward digit span, was relatively more important. This dissociation is important because it enables us to link performance on WD-PW trials specifically to the updating function of working memory, rather than to general working memory capacity. It seems likely, therefore, that working memory updating also benefits SL directly by supporting the acquisition of specific word-form knowledge required for WD-PW discrimination. This provides more concrete evidence for Palmer and Mattys’ (2016) findings which indicate that executive resources are recruited during SL. They previously suggested that working memory updating may assist SL by removing and replacing erroneous syllable grouping from working memory, leading to the more accurate representations required to distinguish words from other familiar-sounding sequences.

Performance on the easier WD-NW trials was less affected by age. This finding is consistent with the results of our regression analysis which revealed that working memory storage capacity, as measured by forward digit span, rather than working memory updating, was the strongest predictor of performance on these trials—note that the older adults performed no worse than the young adults on measures of working memory capacity. It seems likely that performance on WD-NW trials relied more on a general feeling of familiarity with test sequences than on computation of transitional probability. Interestingly, within the recognition memory literature, it has frequently been reported that older adults show an age-related decrease on measures of recollection, but not on familiarity (e.g., Anderson et al., 2008; Koen & Yonelinas, 2016). Impaired recollection in older adults has been linked specifically to impairments in working memory updating (Boujut & Clarys, 2016; Clarys, Bugaiska, Tapia, & Baudouin, 2009), which involves not so much retrieval of content (i.e., feeling of familiarity) as a process of binding and unbinding information. An efficient binding-updating process is likely to underlie word extraction during SL, and therefore performance on WD-PW trials. Since updating is relatively less important on WD-NW trials, we suspect that performance on these trials may depend on recently stored representations, which are not necessarily accurate representations of the words in the stream, but yet provide sufficient information to distinguish between a syllable combination that was heard in the stream from one that was not.

In this study, we observed no reliable effect of speech rate in any of the conditions, except for the cognitive load condition in the older age group on WD-NW trials. This is at odds with the results of our previous study (Palmer & Mattys, 2016), in which we found that a slow rate improved SL performance, presumably because the slower rate afforded more time for working memory processes to contribute to SL. The absence of a clear rate effect in the no-load condition of the current study is therefore intriguing. However, an important difference between this study and Palmer and Mattys’ is that this study included mixed-length (trisyllabic and quadrisyllabic) words, whereas our previous study included only words of uniform length (all trisyllabic). It is possible that the inclusion of mixed-length and/or longer words in the stream made it more difficult to bind syllables into units because of the listener’s inability to predict standard unit length. Weakly bound syllables in the present study could have been particularly sensitive to trace decay intrinsic to slow presentation rates (cf., Baddeley & Lewis, 1984). Thus, with a mixed-length design, any rate-related benefit due to extra processing time could have been counteracted by rate-related decay. Some evidence supporting this line of reasoning can be found in the SRT literature, where slowing down the rate of sequence presentation has been shown to impair implicit learning (Frensch & Miner, 1994). Also, contrary to the findings of Palmer and Mattys (2016), Emberson, Conway, and Christiansen (2011) showed that a slow rate was detrimental to SL in the auditory domain, but beneficial in the visual domain. While the reason for these contradictory findings is not entirely clear, we suspect that the rate effect in SL may rest on a delicate balance between working memory maintenance mechanisms, the level of activation of units held in memory, and the rate of decay. A systematic investigation is required to establish the precise conditions under which slowing down presentation rate stops benefiting performance and instead becomes a hindrance.

Note that the older adults tested in this study did not differ from the younger adults on general measures of working memory capacity (i.e., forward and backward digit span). Our finding that older adults experienced more difficulty with WD-NW trials in the slow rate compared with the normal rate may therefore be linked to the age-related binding deficit which has been widely reported in the aging and memory literature (Chalfonte & Johnson, 1996; Naveh-Benjamin, 2000; Naveh-Benjamin et al., 2003, 2004; Sander, Lindenberger, & Werkle-Bergner, 2012). Within the context of SL, this binding deficit would manifest as an increased difficulty in grouping recurring adjacent syllables into units, which would be magnified by the slower rate, and become observable under cognitive load, where the resources required to actively maintain units held in memory are depleted. The lack of a comparable rate effect for WD-PW trials in older adults is most likely due to a floor effect, since our older participants performed at chance on these trials in both the slow and normal-rate conditions.

To conclude, while SL appears to be relatively resilient to aging when performance is averaged across all conditions, the oldest age group showed some weakness on more sensitive measures of SL, namely, performance on WD-PW trials. This pattern seems to be linked to an age-related decline in working memory updating, which was a strong predictor of performance on these trials. Performance on WD-NW trials, on the contrary, seemed to be largely immune to age-related decline. A distinction between the two trial types was also manifest in the neuropsychological data: While working memory updating predicted performance on WD-PW trials, working memory storage capacity was more important for performance on WD-NW trials. This suggests that global measures of working memory capacity or executive function may be inadequate for assessing the contribution of working memory to SL, which may explain why reliable links between working memory and SL have not been reported in the literature. While this study provides an important initial step in exploring the trajectory of SL over the life span, a number of important issues remain to be addressed. For example, it will be important to determine the effect that explicit task instructions have on older adults’ performance in SL tasks. Future research should also aim to establish the specific circumstances under which slowing down the rate of presentation may help or hinder performance in SL tasks.

## Supplementary Material

10.1037/pag0000292.supp

## Figures and Tables

**Table 1 tbl1:** Hit Rates, False Alarm Rates, and d′ Scores on the 2-Back Task for Young, Middle-Aged (MA), and Older Adults (OA) as a Function of Stream Rate

	Normal rate			Slow rate		
Age group	Hits	False alarms	*d*′	Hits	False alarms	*d*′
Young	.56 (.13)	.03 (.02)	2.14 (.46)	.54 (.17)	.03 (.02)	2.19 (.55)
MA	.59 (.13)	.03 (.03)	2.18 (.36)	.62 (.15)	.03 (.02)	2.31 (.37)
OA	.49 (.14)	.03 (.03)	1.99 (.54)	.50 (.15)	.03 (.06)	2.07 (.39)
*Note.* Standard deviation in parentheses.						

**Table 2 tbl2:** Mean Scores for Young, Middle-Aged (MA), and Older Adults (OA) on Each of the Neuropsychological Tests, Along With F and p Values for Between-Group Comparisons

Tests	Young	MA	OA	*F*	*p*
Hearing	−1.31	7.07	19.66	54.12	<.001***
FDS	6.62	7.40	6.72	4.77	.01*
BDS	5.00	5.58	5.46	1.65	.20
Updating	.82	.82	.73	2.12	
Stroop	−55.27	−101.01	−102.92	7.23	<.001***
Processing speed	45.47	38.25	31.12	28.53	<.001***
*Note.* Hearing = hearing thresholds averaged across all frequencies (dB); FDS = forward digit span; BDS = backward digit span. Updating measured as proportion of letters correct; Stroop measured as the difference between correct and incorrect trials in ms.					
* *p* < .05. *** *p* < .001.					

**Table 3 tbl3:** Bivariate Correlation Coefficients Between Performance on Each of the Neuropsychological Tests and SL Performance Collapsed Across All Conditions (SL AVG), or Split by Trial Type (WD-PW vs. WD-NW) and Cognitive Load (No Load vs. Load)

Tests	SL AVG	WD-PW no load	WD-PW load	WD-NW no load	WD-NW load
Age	−.10	−.15	−.16	.05	−.15
Hearing	−.15	−.16	−.17	−.04	−.11*
FDS	.32**	.16	.22**	.21*	.33**
BDS	.18	.16	.17	.11	.19
Updating	.29**	.24*	.25*	.19	.30**
Stroop	.12	.12	.05	.05	.12
Processing speed	.13	.15	.06	.18	−.07
*Note.* SL= statistical learning; WD-PW = word-partword; WD-NW = word-nonword; Hearing = hearing thresholds averaged across all frequencies (dB); FDS = forward digit span; BDS = backward digit span.					
* *p* < .05. ** *p* < .01.					

**Table 4 tbl4:** Results of the Hierarchical Regression Analysis With SL Performance on the WD-PW Trials as the Dependent Variable

Variance explained	Predictor	β	*p*
Block 1: *R*^2^ = .05, *p* = .09	Hearing	−.11	.50
	Age	−.14	.41
Block 2: *R*^2^ = .19, *p* = .02*	Hearing	−.02	.93
Δ*R*^2^ = .13, *p* = .03*	Age	−.25	.20
	FDS	.14	.30
	BDS	.05	.73
	Updating	.25	.03*
	Stroop	−.07	.51
	Processing speed	−.11	.38
*Note.* SL = statistical learning; WD-PW = word-partword; Hearing = hearing thresholds averaged across all frequencies (dB); FDS = forward digit span; BDS = backward digit span.			
* *p* < .05.			

**Table 5 tbl5:** Results of the Hierarchical Regression Analysis With SL Performance on the WD-NW Trials as the Dependent Variable

Variance explained	Predictor	β	*p*
Block 1: *R*^2^ = .01, *p* = .78	Hearing	−.04	.82
	Age	−.04	.80
Block 2: *R*^2^ = .16, *p* = .04*	Hearing	.03	.87
Δ*R*^2^ = .16, *p* = .01*	Age	−.05	.80
	FDS	.30	.04*
	BDS	−.08	.57
	Updating	.20	.10
	Stroop	.09	.44
	Processing speed	.14	.30
*Note.* SL = statistical learning; WD-NW = word-nonword; Hearing = hearing thresholds averaged across all frequencies (dB); FDS = forward digit span; BDS = backward digit span.			
* *p* < .05.			

## Footnotes

1 Due to technical difficulties, data from the following tasks were unavailable: 2-back data for one older adult in both the slow and normal-rate conditions, and for one middle-aged adult in the slow rate condition; Stroop data for one young adult.
