# Supplementary material for: Statistical Learning for Speech Segmentation: Age-Related Changes and Underlying Mechanisms
Source: Psychol Aging. 2018 Sep 24;33(7):1035–44. doi: 10.1037/pag0000292 (PMC6233520; doi:10.1037/pag0000292)
Supplement: Supplementary file 1 [file PAG-2017-1302Suppl.zip › Online Supplement.docx]

**Online Supplement**

 *Figure 1.* Hearing thresholds for young, middle-aged, and older adults averaged across left and right ears. Error bars represent the standard error of the mean.

**Stimuli**

Table 1: Words, Part-words, and Nonwords used in the test phase for each of the four languages.

**_________________________________________________________________________**

Language Stream A words Stream B words Nonwords

(Stream B part-words) (Stream A part-words)

________________________________________________________________________

1 lasokachu bufilaso chuvibugo

pemadovi nugopema finukado

tinugo kachuti matipe

rebufi dovire sorela

_________________________________________________________________________

2 pavuchiga senopavu banogali

mikeroba litumike turochise

duseno robadu kedumi

folitu chigafo vufopa

__________________________________________________________________________

3 bisamuko chevobisa kochenad

lutaderi fenaluta rifevomu

pofena deripo sapali

gucheveo mukoga tagubi

__________________________________________________________________________

4 kuchavemo rafokucha fovegito

dapilogi tosudapi mosuralo

nirafo vemoni chaniku

betosu logibe pidabe

__________________________________________________________________________

**Neuropsychological Tests**

*Digit Span tasks*

The forward and backward digit span tasks (FDS and BDS, respectively) each consisted of 24 trials. All participants performed the FDS task before the BDS task. For both tasks, each trial consisted of a sequence of digits presented auditorily. After each sequence, participants were required to repeat the digits in the same order (FDS) or reverse order (BDS). They then pressed the space bar to begin the next trial. The number of digits in each sequence increased by one every three trials, starting with three digits, and ending with a maximum of 10 digits. The test was terminated when the participant made a total of three errors. A trial was scored as correct if all digits were repeated in the correct serial position. No feedback was provided. Digit span was calculated by averaging the number of digits in the last three trials that the participant had repeated correctly.

*Working Memory Updating Task*

Working memory updating was assessed using an adapted version of the Letter Memory task (Morris & Jones, 1990) following the procedure of St Clair-Thompson and Gathercole (2006). In this task, participants are presented with a sequence of consonants on a computer screen, one at a time. Each consonant appeared for 2 seconds, and the number of consonants in each sequence varied, unpredictably, on a trial by trial basis (between 5 and 11 consonants). Participants were asked to rehearse the last 4 consonants in each sequence. Since participants did not know how many consonants would be presented ahead of each trial, they were told that they would need to update the content of their memory in order to perform the task. At the end of the sequence, a question mark appeared on the screen, and the participant was required to write down the last four consonants in the sequence. Working Memory updating was calculated as the number of letters recalled correctly. To be scored as correct, the letter has to appear in the correct serial position.

*Stroop task*

The Stroop task included 72 trials (24 congruent, 24 incongruent, and 24 neutral) split into three blocks, with a self-paced break period between blocks. The stimuli consisted of four colour words (red, green, blue, and yellow), which each appeared 6 times in a congruent ink colour and 6 times in an incongruent ink colour. Four number words (six, eight, nine, and twelve) were used as stimuli for the neutral trials. Those number words were selected because they matched the colour words in both length and frequency. Each number word appeared 6 times in one of the four ink colours, and each ink colour was represented an equal number of times in the neutral trials. All stimuli were presented on a computer monitor with a black background. Each trial began with a white fixation cross (500 ms) followed by a blank screen (200 ms). Then, one of the words appeared and remained on the screen for 2000 ms. Participants were instructed to name the colour of the ink in which the word was presented as quickly and accurately as possible, ignoring the meaning of the word. Response times were measured from the onset of the visual presentation to the initiation of the spoken response.
